# Supplementary material for: Splice-Junction-Based Mapping of Alternative Isoforms in the Human Proteome
Source: Cell Rep. Author manuscript; Available in PMC 2020 Jan 15. (PMC6961840; doi:10.1016/j.celrep.2019.11.026)
Supplement: 2 [file NIHMS1546469-supplement-2.zip › DF1/PXD010154/Testis/Testis_38_KANSL2_ASAPLSQSGLATANGKPEPTSIPYHSFQSEVLGLCGPTNR.pdf]

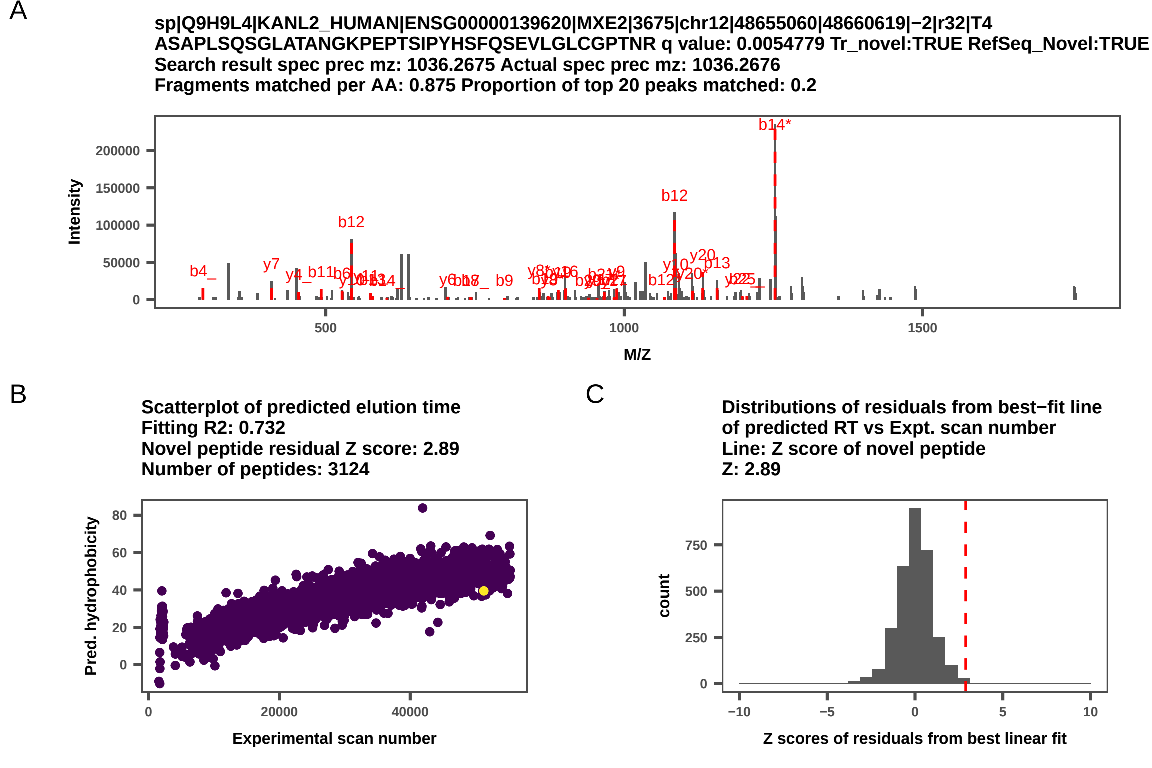
A
sp|Q9H9L4|KANL2_HUMAN|ENSG00000139620|MXE2|3675|chr12|48655060|48660619|−2|r32|T4
ASAPLSQSGLATANGKPEPTSIPYHSFQSEVLGLCGPTNR q value: 0.0054779 Tr_novel:TRUE RefSeq_Novel:TRUE
Search result spec prec mz: 1036.2675 Actual spec prec mz: 1036.2676
Fragments matched per AA: 0.875 Proportion of top 20 peaks matched: 0.2
Intensity
200000
150000
100000
50000
0
b14*
b12
b12
b4_
y7
y20
b13
b9
M/Z
500
1000
1500
B
Scatterplot of predicted elution time
Fitting R2: 0.732
Novel peptide residual Z score: 2.89
Number of peptides: 3124
Pred. hydrophobicity
80
60
40
20
0
0
20000
40000
Experimental scan number
C
Distributions of residuals from best−fit line
of predicted RT vs Expt. scan number
Line: Z score of novel peptide
Z: 2.89
count
750
500
250
0
−10
−5
0
5
10
Z scores of residuals from best linear fit
